# Cross-cousin marriage among Tsimane forager–horticulturalists during demographic transition and market integration

**DOI:** 10.1017/ehs.2024.11

**Published:** 2024-03-26

**Authors:** Arianna Dalzero, Bret A. Beheim, Hillard Kaplan, Jonathan Stieglitz, Paul L. Hooper, Cody T. Ross, Michael Gurven, Dieter Lukas

**Affiliations:** 1 Department of Human Behavior, Ecology and Culture, Max Planck Institute for Evolutionary Anthropology, Leipzig, Germany; 2 Economic Science Institute, Chapman University, Orange, California, USA; 3 Toulouse School of Economics and Institute for Advanced Study, University of Toulouse 1 Capitole, Toulouse, France; 4 Department of Anthropology, University of New Mexico, Albuquerque, New Mexico, USA; 5 Department of Anthropology, University of California, Santa Barbara, California, USA

**Keywords:** Cousin marriage, life-history, demography, kinship, Tsimane

## Abstract

Although still prevalent in many human societies, the practice of cousin marriage has precipitously declined in populations undergoing rapid demographic and socioeconomic change. However, it is still unclear whether changes in the structure of the marriage pool or changes in the fitness-relevant consequences of cousin marriage more strongly influence the frequency of cousin marriage. Here, we use genealogical data collected by the Tsimane Health and Life History Project to show that there is a small but measurable decline in the frequency of first cross-cousin marriage since the mid-twentieth century. Such changes are linked to concomitant changes in the pool of potential spouses in recent decades. We find only very modest differences in fitness-relevant demographic measures between first cousin and non-cousin marriages. These differences have been diminishing as the Tsimane have become more market integrated. The factors that influence preferences for cousin marriage appear to be less prevalent now than in the past, but cultural inertia might slow the pace of change in marriage norms. Overall, our findings suggest that cultural changes in marriage practices reflect underlying societal changes that shape the pool of potential spouses.

## Graphical abstract

**Figure:**
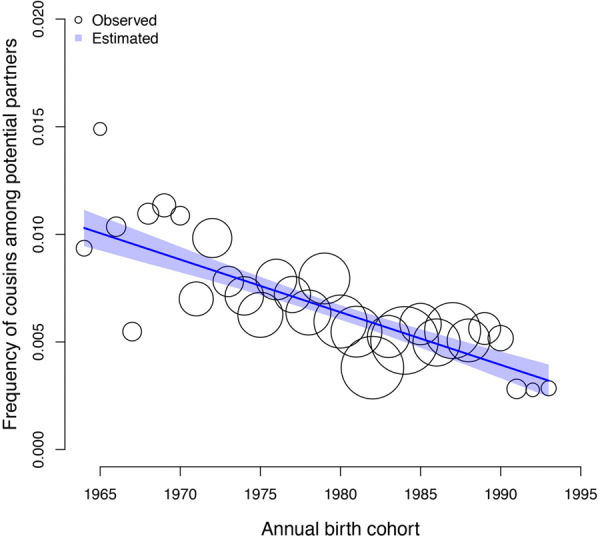

**Social media summary:** Traditions in a changing world: partner availability, fitness effects and the decline in cousin marriage among Tsimane.

## 1. Introduction

Many human populations are experiencing rapid demographic, socioeconomic and cultural changes, including demographic transition (Borgerhoff Mulder, 1998), industrialisation, market integration and urbanisation (Mattison & Sear, 2016). These changes affect – and are affected by – family composition and norms governing spousal partner choice. Cousin marriage – a spousal union between individuals who share one or more grandparents – used to be practised at high rates in many human societies (Shaw & Raz, 2015). It is still estimated to account for more than 10% of spousal unions worldwide (Bittles & Black, 2010), but its frequency has been declining in many societies undergoing demographic and socioeconomic transitions (Sahoo et al., 2022; Brennan, 1981; Kalam et al., 2021). This has led some to forecast a global decline in cousin marriages (Bittles & Black, 2010). Changes in the frequency of cousin marriage are often associated with changes in other cultural norms – e.g. owing to particular religious proscriptions (Cazes, 1996; Schulz et al., 2019) – but can also reflect broader changes in demographic and socioeconomic circumstances that might influence marriage decisions indirectly (Shenk et al., 2016). More specifically, changes in demographic and socioeconomic circumstances have often been shown to co-occur with changes in the frequency of cousin marriage (Peña et al., 2002; Givens & Hirschman, 1994; Assaf & Khawaja, 2009; Kumari et al., 2020; Shenk et al., 2016). Demographic circumstances, for example, are expected to shape the pool of potential marriage partners (Hajnal, 1963). Socioeconomic circumstances might also influence the potential fitness returns to particular marriage decisions (Ross et al., 2018), and thus affect people's preferences for them (Borgerhoff Mulder & Ross, 2019; Micheletti, 2020). In this paper, we investigate whether – and how – changes in the occurrence of first cousin marriage are associated with changes in the demographic composition of the pool of eligible partners and the potential fitness returns to marrying a first cousin. We focus our case study on the Tsimane forager–horticulturalists of the Bolivian Amazon. While we consider socioeconomic factors like wealth, social network structure and residence patterns in interpreting these dynamics, our analysis does not directly delve into modelling such factors, owing to a lack of data (see Study Limitations).

A central question raised by researchers studying cousin marriage is whether individuals choose their spouses based on different characteristics and/or simply search for potential partners from a broader pool of individuals during periods of demographic and socioeconomic changes (e.g. owing to globalising forces) (Goode, 1963; Givens & Hirschman, 1994). The prevalence of cousin marriage can be high in societies where the number of suitable partners is limited – e.g. in geographically isolated populations – and studies have shown that the availability of cousins as partners influences partner choice (Mobarak et al., 2019; Barakat & Basten, 2014). In such circumstances, individuals may choose cousins as partners in order to avoid the costs of migration to find a mate and to increase their marriage opportunities (see below for details on the potential fitness benefits of cousin marriage) (Hoben et al., 2016; Cavalli-Sforza et al., 2013). An increase in social connections between isolated populations and larger population centres is often associated with a decrease in the frequency of cousin marriage (Pettener, 1985). Past studies have thus hypothesised a causal connection between changes in the frequency of cousin marriage over time and shifts in the composition of the pool of potential mates (Givens & Hirschman, 1994; Helgason et al., 2008). In particular, past work has linked early periods of demographic transition to an increase in fertility and a decrease in childhood mortality rates – circumstances that may lead to greater availability of kin among potential partners (Givens & Hirschman, 1994; Peña et al., 2002; Pettener, 1985; Murphy, 2011; Brennan, 1981). These studies, however, are generally focused on closed populations or on short-term outcomes, and it is unclear how the pool of eligible partners might change in the long term, and in more interconnected, growing populations, where changes in the number of unrelated potential partners may exceed changes in the number of kin (Lukas et al., 2005).

Socioeconomic shifts – such as market integration – might have differential impacts on the fitness outcomes of cousin vs. non-cousin marriages – e.g. through differences in moderation of the direct biological effects of marriage practice or through impacts on the socioeconomic factors that influence marriage partner choice (Mobarak et al., 2019; Dalzero et al., 2023). Changes in fitness returns to cousin marriage might in turn cause shifts in individuals’ preferences to marry a cousin. Fitness outcomes and the frequency of cousin marriage are closely tied to the particular socioeconomic conditions of a population. Cousin marriage rates are notably high in specific societies – especially agricultural and horticultural ones – where it offers advantages like increased access to kin-based wealth (Johow et al., 2019) and alliances (Lévi-Strauss, 1971), greater opportunities for marriage, and a smoother and faster marriage process through matrimonial exchanges and agreements (Hoben et al., 2016; Cavalli-Sforza et al., 2013; Chagnon et al., 2017). All of these factors can contribute to fitness gains – e.g. through increased fertility and offspring survival, and earlier age at reproduction (Bailey et al., 2014; Chagnon et al., 2017; Mobarak et al., 2019; Dalzero et al., 2023). Fitness gains may offset the potential cost arising from the biological consequences of mating with close relatives (Clark et al., 2019). Preferences for cousin marriage have indeed been shown to increase in association with dowry and land ownership systems that prioritise family assets (Peña et al., 2002). In contrast, declines in cousin marriage have also been linked to economic changes that facilitate more resource independence for women – e.g. increased levels of education, availability of wage labour and engagement in a market economy appear to attenuate the frequency of cousin marriage, rendering intensive kinship systems and kin-based alliances less beneficial (Assaf & Khawaja, 2009; Bras et al., 2009; Islam, 2018; Brennan, 1981; Koc, 2008; Shenk et al., 2016; Schaffnit et al., 2022).

Importantly, people practising cousin marriage might also be in different socioeconomic conditions than people not practising it – e.g. in terms of residence, social and kin networks, and wealth – and market integration might affect these conditions (Ghosh et al., 2023). Socioeconomic changes are also expected to alter the trade-offs between different components of fitness in cousin and non-cousin marriages. For example, if a high fertility rate and early age at reproduction trade off against child or maternal survival, this relationship is likely to be influenced by shifts in healthcare or reproductive behaviours (Davison & Gurven, 2021). Recent literature on cross-cultural patterns in cousin marriage practices documents the correlates (e.g. industrialisation, dowry, and cultural traditions) of changes in the frequency of cousin marriage, especially as they relate to economic development (Shenk et al., 2016; Bittles & Black, 2010; Caldwell et al., 1983). However, most studies in the literature were not able to determine how changes in partner choice relate to realised differences in fitness returns, as such analyses require data on the costs and benefits of different marriage strategies in terms of both parent reproduction and offspring survival. Likewise, studies that have examined the relationship between kinship and fertility – or offspring survival – in human populations (Mobarak et al., 2019; Helgason et al., 2008; Husain & Bunyan, 1997; Hussain & Bittles, 2004; Bittles et al., 2002) did not test for temporal dynamics in these fitness components for cousin and non-cousin marriages. We hope to fill in these gaps in understanding using data from a large, longitudinal sample of individuals from the Tsimane forager–horticulturalists of the Bolivian Amazon.

The Tsimane are a high-fertility, isolated population with limited use of contraception. Tsimane cultural practices feature cross-cousin marriage – where a focal man marries the daughter of his mother's brother or his father's sister. Traditionally, Tsimane stigmatise those who do not marry a cross-cousin, who – according to local legend – are believed to become jaguars after they die (Godoy et al., 2008). While marrying a first cross-cousin (*fom cheyas*) is considered ideal, in cases when first cousins are not available, the second option is to marry a more distant cousin in the cross lineage (*fom moch*) (Ellis, 1997). In demographic interviews conducted between 2002 and 2005 in 18 villages across the Tsimane territory (M. Gurven, unpublished), 64 couples (31%) reported being first cross-cousins, 92 couples (45%) reported being distant cross-cousins and 48 couples (24%) reported being unrelated. Similarly, other estimates suggest that the frequency of cross-cousin marriage (including first and distant cousins) is 75% in some areas (unpublished pilot survey reported in Patel et al., 2007). Opposite-sex siblings often arrange marriages between their respective children (Winking et al., 2011), or otherwise exert informal pressure on their children's choice of partners (Godoy et al., 2008). However, marriages are often decided by the couple, in accordance with the custom of preferential cross-cousin marriage. Tsimane marriage partners are generally similar to each other in age and wealth. Tsimane do not commemorate marriages with formal ceremonies, but rather consider a pair to be married when they sleep together in the same house. Newlyweds reside with the wife's family until the birth of their first child, at which point they typically transition to a neolocal residence (Godoy et al., 2008). The median age of marriage among Tsimane is 21 for males and 16.5 for females. Women, on average, become mothers by the age of 18 (Martin & Gurven, 2022). The Tsimane are primarily endogamous, occasionally intermarrying with lowland groups who have settled in Tsimane territory (Pisor & Gurven, 2018). Monogamy is the norm among Tsimane (95% of married men, with 5% practising sororal polygyny), and divorce is rare.

The Tsimane are undergoing a socioeconomic shift from a horticultural subsistence economy to a more market-integrated economy, and simultaneously experiencing a demographic transition (Gurven et al., 2017). Both of these factors may influence the availability of potential partners, the choices individuals make in selecting partners, and individuals’ survival and reproduction. Most Tsimane still subsist on slash-and-burn horticulture, fishing and hunting. However, owing to an agrarian reform in 2006 that distributed forests to highland colonist farmers, they are becoming increasingly dependent on cash cropping, selective logging and/or on employment in logging camps, cattle ranches and farms (Reyes-García et al., 2014). The Tsimane are in the first phase of a demographic transition, with an annual population growth rate of over 3.5% (Gurven et al., 2017). This population growth results from a combination of reduced child and adult mortality, owing to increased access to healthcare facilities, and individuals maintaining a preference for high fertility, with women conceiving an average of nine children over their life course (Kaplan et al., 2015; Mcallister et al., 2012). While high fertility and the fall of death rates may lead to increases in the number of cross-cousins available as potential partners (Givens & Hirschman, 1994), this increase in number of kin may be outpaced by increases in the number of unrelated individuals available as potential partners, owing to the overall growth of the population (Lukas et al., 2005), especially in more connected areas (Pettener, 1985; Brennan, 1981).

In fact, while Tsimane have traditionally lived in small dispersed settlements, often connected by rivers, and mainly composed of extended family clusters, they now tend to aggregate in larger settlements, closer to market towns, roads and logging camps (Gurven et al., 2017). Changes in residence and movement patterns may lead to an increase in contact with unrelated potential partners. Traditionally, beginning in adolescence, Tsimane men often travel to visit relatives in other villages. Many young men use these frequent visitations to relatives as an opportunity to seek their future spouses among their cross-cousins and to obtain permission for the marriage from future in-laws (Miner et al., 2014). According to Godoy et al. (2007), 79% of individuals find their spouse in a different village. In contrast, members of more connected and larger communities travel more often to market towns (Miner et al., 2014), possibly expanding their social networks and increasing their chances of establishing relationships with non-kin. As a consequence of migration and re-settlement, changes in kin-based and broader sharing networks might also affect the potential fitness benefits of marrying a cross-cousin (Hooper et al., 2015; Sear & Coall, 2011). Tsimane sharing networks are strongly shaped by kinship (Hooper et al., 2015). However, the access to family wealth and alliances that might have traditionally increased the reproductive success of people practising cousin marriage might be becoming less important (Borgerhoff Mulder et al., 2009; Johow et al., 2019). With increasing reliance on a cash and wage-based economy, factors other than kin ties might be more relevant for partner choice – for example, individuals who speak Spanish might be becoming more attractive partners because of their ability to communicate with individuals in the wider market economy, thus providing better access to resources important for family well-being and reproductive success (Godoy et al., 2007). Access to modern medicine, and the resulting reduction in mortality (Gurven & Kaplan, 2007), might also reduce differences in fitness between families practising different marriage strategies. In these novel circumstances, individuals might be expected to choose their partners based on new, more personal criteria, rather than adhering to cultural norms or family preferences. Such changes in choice might be further facilitated by a breakdown of traditional and parental authority (Reyes-García et al., 2014; Godoy et al., 2008).

Our focus here is to understand whether normative first cross-cousin marriage might have become less attractive over time in this population, potentially because of changes in the composition of the pool of eligible partners or because of reduced fitness benefits of kin-intensive networks. The detailed long-term data collected by the Tsimane Health and Life History Project (THLHP; Gurven et al., 2017) afford a unique opportunity to unravel the dynamics of cousin marriage in a changing society. We rely solely on the THLHP genealogical data, because these data span the period of time in which the Tsimane livelihood began to transition from horticultural subsistence to market integration, and include individuals born from the end of the nineteenth century to the beginning of the twenty-first century. We begin by quantitatively assessing the extent to which the frequency of first cross-cousin marriage has changed over this several decade period. We then investigate the role of partner availability on the occurrence of first cross-cousin marriage. Specifically, we investigate whether the composition of the pool of potential partners has changed over time, and whether such changes can explain changes in the frequency of first cross-cousin marriage. Next, we explore the age-specific fitness costs and benefits of first cross-cousin marriage for both women and men, by comparing life history traits (i.e. offspring child survival rates, fertility rates, and ages at first reproduction) that might be expected to differ between families practising cousin marriage and families not practising it (Dalzero et al., 2023; Bailey et al., 2014). The joint estimation of these life history traits in demographic models can potentially identify trade-offs that might occur. Finally, we examine whether any patterns in these life-history traits have changed over time. Table 1 provides an overview of our questions, and inferential estimands.

**Table 1.** Research questions and inferential estimands

Question	Inferential estimand
Has the frequency of cousin marriage changed over time?	Frequency of cousin marriage as a function of the birth cohort of individuals.
Is cousin marriage among Tsimane limited by the number of cousins available as eligible partners?	Frequency of cousin marriage as a function of the number of cousins available as potential partners.
Is cousin marriage among Tsimane limited by the frequency of cousins in the pool of potential partners?	Frequency of cousin marriage as a function of the frequency of cousins in the pool of potential partners.
Do Tsimane marry cousins more than expected by chance in relation to potential partners?	Frequency of cousin marriage as a function of expected frequency based on frequency of cousins.
Has the frequency of cousins in the pool of potential partners changed over time?	Change in frequency of cousins in the pool of potential partners as a function of birth cohort.
Is the change in frequency of cousin marriage over time associated with the change in the frequency of cousins in the pool of potential partners?	Frequency of cousin marriage as a function of the frequency of cousins in the pool of potential partners and birth cohort.
Do individuals married to cousins have costs and/or benefits in terms of offspring survival?	Difference in survival probability up to age 5 between children born to cousins and born to non-cousins.
Do individuals married to cousins have costs and/or benefits in terms of fertility?	Difference in age-specific fertility between individuals married to a cousin and individuals married to a non-cousin partner.
Do individuals who marry cousins have costs and/or benefits in terms of marriage opportunities?	Difference in age at first reproduction between individuals married to a cousin and individuals married to a non-cousin partner.
Has the difference in child survival between offspring of cousins and non-cousins changed over time?	Difference in probability of survival up to age 5 between birth cohorts of children born to cousins and born to non-cousins.
Has the difference in fertility between individuals married to cousins and to non-cousins changed over time?	Difference in number of children between birth cohorts of individuals married to cousins and to non-cousins.
Has the difference in marriage opportunities between individuals who marry cousins and non-cousins changed over time?	Difference in age at first reproduction between birth cohorts of individuals married to cousins and to non-cousins.

## 2. Methods

### 2.1. Data source

The THLHP has been conducting genealogical and reproductive history interviews in the Tsimane territory since 2002. The study encompasses 90 villages that are almost exclusively occupied by Tsimane people. The Tsimane population register contains data at the individual level, spanning a period from 1870 to 2014, and currently describes 28,093 individuals. The register includes information on the unique ID, gender, birth date and death date for each individual. For 30% of the register, there is additional data on the biological and socially recognised parents of each focal individual.

### 2.2. Data preparation and relatedness classification

We use genealogical information to classify marriage types. We test if individuals are in cousin marriages by assessing if individuals who are partners have at least one grandparent in common. In order to distinguish (first degree) cross-cousins (hereafter referred to as ‘cousins’) from parallel-cousins, we checked for any shared maternal and paternal grandparents in each couple. We excluded a small number of parallel cousins (four marriages) from our empirical analyses, and we excluded parallel cousins from the set of eligible partners, because parallel cousin marriage is considered incestuous among Tsimane. Since information on great-grandparents is unavailable for the majority of couples, we classify as cross-cousins only those who are first-degree cross-cousins – i.e. the individuals referred to as ‘*fom cheyas*’ in the Tsimane language. Distant cousins (‘*fom moch*’) are categorised as non-cousins (see Study Limitations). As such, our inferences here are specifically limited to patterns in first degree cross-cousin marriage.

In order to identify couples, we assume that two individuals are married if they share parentage for a child, as we lack specific information on marriages *per se* (see the section ‘Study limitations’). We test if marriages are between first cousins using two different relatedness classification techniques. Our ‘relaxed’ condition classifies all marriages in which at least one of the eight grandparents are known for each partner. Using this classification technique, we find 546 individuals married to cousins, 4217 individuals married to non-cousins, 1230 individuals born to cousins and 12081 individuals born to non-cousins. Because some grandparents are unknown, however, this approach necessarily under-estimates the proportion of cousin marriages (see the section ‘Study limitations’). Our ‘strict’ relatedness classification, in contrast, only includes marriages in which all eight grand-parents are known. Using this technique, we find 319 individuals married to cousins, 808 individuals married to non-cousins, 607 individuals born to cousins and 1920 individuals born to non-cousins. Thus, between 11 and 28% of Tsimane marriages – for which assessment is possible – are between first degree cross-cousins. We use the ‘strict’ relatedness classification for the analyses on the frequency of cousin marriage and eligible partners (questions 1–3), to avoid classifying people as not having cousins available as potential partners just because their grandparents are unknown. We use the ‘relaxed’ relatedness classification for the analyses assessing age-specific fitness and change in fitness over time (questions 4 and 5), in order to increase sample size, especially among individuals born longer ago. The absence of information about grandparents is less problematic in the fitness analyses because we do not need to identify potential cousin partners here (see the section ‘Study limitations’). Repeating the fitness analyses with the sample of individuals classified using the ‘strict’ relatedness criterion leads to qualitatively similar results.

In order to estimate the demographic composition of the pool of potential partners for each focal person, we first calculate the age difference between partners who are in unions – i.e. people who are classified as mothers or fathers of the same children – and summarise this range using the 90% HPDI (highest posterior density interval). Next, we calculate the number of potential eligible cousin and non-cousin partners (excluding the parallel cousins) for each individual at the age when they have their first child. Using the age at the first child as a proxy for when an individual forms a partnership, we rely on the observation that there is a relatively short time gap between the formation of marital union and the birth of the first child among Tsimane. The ‘potential partners’ of each individual are defined by assessing the number of unmarried opposite sex individuals who are within an appropriate age range, based on the empirical distribution. Male potential partners can be up to 9 years older or 1 year younger than a focal woman, and female potential partners can be up to 9 years younger or 1 year older than a focal man. We include individuals who are already married (either to cousins or not) in the analyses of the frequency of cousin marriage and eligible partners (questions 1–3) and in the analyses of fertility and age at first reproduction (questions 4.2, 4.3, 5.2 and 5.3). To estimate fertility for each marriage type, we count the number of offspring of each individual and note their age at each of their births (AOB). Similarly, to estimate offspring survival for each marriage type, we note the age of death (AOD) or age of censoring (AOC) for each child – the AOC represents the age of individuals when they were last sampled, if they are still living.

### 2.3. Data analyses

For the first question (change in the frequency of cousin marriage over time), we estimate the association between marriage type and annual birth cohort. For the second group of questions (frequency of cousin marriage in relation to the set of eligible partners), we estimate the association between marriage type and the number of cousins and non-cousins available as partners, as well as the association between marriage type and the frequency of cousins in the pool of potential partners (i.e. the number of cousins divided by the number of cousins and non-cousins available as potential partners). For the third group of questions (change in the pool of eligible partners over time), we estimate the association between the frequency of cousins in the pool of potential partners and the annual birth cohort of individuals. Next, we estimate the association between marriage type and the frequency of cousins in the pool of potential partners, controlling for secular trends owing to annual birth cohort. We use annual birth cohort (i.e. birth year) to measure change in the frequency of cousin marriage and change in the pool of potential eligible partners over time (questions 1–3). For the analyses including annual birth cohort, we restricted inference to the years in which at least 10 individuals with known marriage relation were recorded – i.e. the period between 1964 and 1993.

For the fourth group of questions (age-specific fitness), we estimate age-specific probabilities of offspring survival by marriage type. We also estimate age-specific probabilities of reproduction by marriage type. For these analyses, we include individuals born from 1870 to 2014, the full time-span of the database. For the fifth group of questions (change in fitness over time), we estimate the probability of survival from birth to age 5 as a function of decadal birth cohort and parental marriage type. Additionally, we estimate fertility (i.e. number of children) and age at first reproduction, as a function of decadal birth cohort and marriage type. We use decadal birth cohort (i.e. birth decade) to measure change in fitness over time (question 5). We summarise the data by decade in order to capture potential non-linear shifts in key fitness parameters over time. We consider decades of birth in which at least 10 births were recorded for each sub-population (i.e. each group of individuals married or born to cousins or to non-cousins). In the offspring survival model, we include data from 1950 to 2000, and in the fertility and age at first reproduction models, we include data from 1940 to 2000 (see the section ‘Study limitations’).

We perform analysis using R, version 4.3.1 (R Core Team, 2021). We use the rethinking package (version 2.21) to construct all statistical models (McElreath, 2020), apart from the age-specific survival and fertility models, for which we used CmdStan, version 2.33.1, directly (Stan Development Team, 2021; Carpenter et al., 2017). We use the rethinking package in R (McElreath, 2020) to summarise the results. All the code needed to reproduce our analyses will be maintained at: https://doi.org/10.17605/OSF.IO/PK8F7. Individual-level data are stored in the THLHP Data Repository, and are available through protected access protocols (see the ‘Research transparency, reproducibility and data availability’ section). Data were drawn from the THLHP database as of 2 November 2021. For more information, please see: https://tsimane.anth.ucsb.edu/data.html.

### 2.4. Analyses of the frequency of cousin marriage over time

In order to see if the frequency of cousin marriage has changed over time (question 1), we estimate the association between marriage type (i.e. cousin vs. non-cousin marriage) and the annual birth cohort of individuals. We build a logistic regression that is specified by the following set of equations:
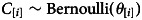

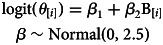


where the response variable, *C*, is a binary indicator of whether an individual ever participated in a cousin marriage, and the predictor variable, *B*, is the annual birth year of each individual (i.e. of individual *i*). The parameter vector *β* gives the estimated coefficients – i.e. the intercept and the slope on annual birth cohort. The slope coefficient on annual birth cohort (*β*_2_) reflects how the frequency of cousin marriage has changed as a logit–linear function of time.

### 2.5. Analyses on frequency of cousin marriage in relation to eligible partners

In order to understand whether cousin marriage among the Tsimane is limited by the number of cousins that are available as potential partners (question 2.1), we estimate the association between marriage type and the number of cousins available as potential partners for each individual. We build a binomial logistic regression specified by the following set of equations:
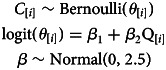
where the response variable, *C*, is a binary indicator of cousin marriage as above, and the predictor variable, *Q*, is the number of cousins available as potential partners for each individual (i.e. for individual *i*). The slope coefficient on number of cousins (*β*_2_) reflects how the frequency of cousin marriage covaries with number of cousins.

In order to understand if cousin marriage among the Tsimane is determined by the (median-centred) frequency of cousins in the pool of potential partners of each individual (question 2.2), and whether Tsimane people marry cousins more than expected by chance in relation to their available pool of potential partners (question 2.3), we estimate the association between the individuals’ marriage type and the frequency of cousins in their pool of potential partners. We build a logistic regression specified by the following set of equations:
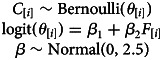
where the predictor variable, *F*, is the frequency of cousins in the pool of potential partners of each individual (i.e. of individual *i*). From this estimation, we extract the slope coefficient – i.e. the *β*_2_ coefficient – to see the effect of the frequency of cousins in the pool of potential partners on the chance to marry a cousin (question 2.2). We extract the intercept – more specifically, the inverse logit of *β*_1_ – to test if Tsimane marry cousins more than expected by chance (question 2.3).

### 2.6. Analyses on the change in the pool of potential eligible partners over time

In order to see if the frequency of cousins in the pool of potential partners has changed over time (question 3.1), we estimate the linear association between the frequency of cousins in the pool of potential partners of each individual and the (median-centred) year of birth of participating individuals. We build a linear regression specified by the following set of equations:
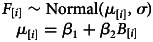

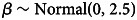

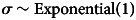
where the response variable *F* is the observed frequency of cousins eligible as partners and the predictor variable, *B*, is the annual birth cohort of each individual. Here, *σ* is a standard deviation parameter. We also calculate the difference in *μ* for the maximum and minimum value of *B*.

In order to understand whether the change in frequency of cousin marriage over time is determined by the change in the frequency of cousins available as partners for individuals born in different years (question 3.2), we estimate the association between marriage type and the frequency of cousins in the pool of potential partners of each individual, controlling for annual birth cohort. We build a binomial logistic regression specified by the following set of equations:
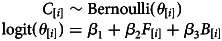

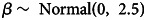


where the response variable, *C*, is a binary indicator of cousin marriage. The parameter vector *β* gives the intercept and slopes indicating the effect of the frequency of cousins as available partners and the effect of annual birth cohort on the response variable.

### 2.7. Analyses on fitness costs and benefits of cousin marriage

In order to assess if individuals married to cousins experience costs and/or benefits in terms of offspring survival (question 4.1), we estimate age-specific survival probabilities (i.e. for each age *x*, the probability of survival to age *x* + 1, given that individuals survived to age *x*), for individuals born to parents who are cousins and for individuals born to parents who are non-cousins. For these analyses, we include individuals born from 1870 to 2014. We build aggregated binomial regressions with varying effects on age categories and type of marriage, specified by the following set of equations:
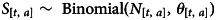
where *S*_[_*_t,a_*_]_ is the number of individuals surviving for each parental marriage type, *t*, and age category, *a*, *N*_[*t,a*]_ is the number of individuals at risk and *θ*_[*t,a*]_ is the survival probability for each parental marriage type and age class. We then model:
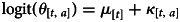
where *μ*_[_*_t_*_]_ is an intercept for given parental marriage type and *κ*_[_*_t,a_*_]_ is an offset for each age category.
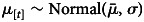


The hyperparameters *μ* and *σ* partially pool information between parental marriage types.
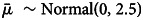

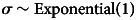


To define the age-category offsets, we use a Gaussian Process approach:
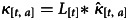
where 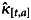 are unit normal random effects and *L*_[*t*]_ is a Cholesky factor from the decomposition of the covariance matrix, Ω_[_*_t_*_]_, which describes variation and covariation among posterior random effects for each age category, for marriage type *t* (McElreath, 2020). The covariance matrix is defined using an exponential decay function in Stan. This model structure allows for the probability of survival to take on arbitrary functional forms with respect to age, while still sharing information across neighbouring age categories. More specifically, the Gaussian process approach: (1) allows for partially pooled estimation of parameters; (2) imposes no *a priori* functional form (e.g. linear, quadratic, etc.) on random effects; and (3) allows for a reduction in parameter complexity of the model by reducing the extent to which neighbouring random effects on age category can vary independently. The Cholesky decomposition formulation is simply a numerically efficient way to parameterise the Gaussian Process model (interested readers should refer to Barnard et al., 2000, for technical details).

We extract the posterior distributions of age-specific survival probabilities, for individuals born to cousins and born to non-cousins, and we use them to reconstruct survival curves. We compute child survival as the product of age-specific survival coefficients up to age 5. We compute the contrasts in child survival between those individuals born to cousin and those individuals born to non-cousins.

We use similar Stan models to assess if individuals married to cousins have costs and/or benefits in terms of fertility (question 4.2) and age at first reproduction (question 4.3). In these cases, the Stan model is used to estimate the probability of reproducing at each age of life for individuals married to partners who are cousins and for individuals married to partners who are non-cousins. The variable *S*_[_*_t,a_*_]_ is therefore replaced with the variable *R*_[_*_t,a_*_]_, which defines the number of individuals reproducing for each marriage type, *t*, and age category, *a*.

To estimate age-specific fertility, and age at first reproduction, we extract the posterior distributions of the probability of reproducing at each age, for individuals married to cousins and individuals married to non-cousins, and we use them to reconstruct the relevant fertility curves. Overall fertility here is defined as the sum of age-specific fertility across reproductive ages. The age at first reproduction here is defined as the first age when the expected number of births exceeds 1. We compute contrasts in fertility and age at first reproduction between each marriage type.

### 2.8. Analyses of temporal trends in fitness costs and benefits of cousin marriage

Here, we measure longitudinal changes in offspring survival, fertility, and age at first reproduction. In order to assess how the chance of surviving up to age 5 has changed over time for offspring of cousin and non-cousin parents (question 5.1), we estimate the chance to survive up to age 5 by parental marriage type for individuals born in each decade, *D*, between 1950 and 2000. We build a fixed-effect logistic regression specified by the following set of equations:
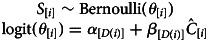

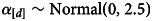

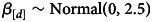
where the response variable, *S*, is a binary indicator for survival up to age 5, 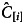 is an indicator for being born to cousins, *α* is a vector of intercept parameters for each decadal birth cohort and *β* is a vector of slopes giving the estimated difference in log-odds of survival up to age 5 between individuals born to cousins and individuals born to non-cousins, for each decadal cohort. Here, and below, the function *D*(*i*) returns the birth decade of individual *i*. We fit this model independently for focal male and female individuals.

In order to assess how the fertility of individuals married to cousins and individuals married to non-cousins changed over time (question 5.2), we estimate longitudinal changes in fertility (for individuals born in decades between 1940 and 2000), using Poisson regression specified by the following set of equations:
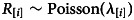

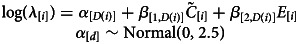

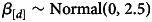
where the response variable *R* is the observed number of children of each individual, *α* is a vector of intercept parameters for each decadal birth cohort, *β*_1_ is a vector of slope parameters for the effect of spousal cousin marriage, *C*˜, on fertility for each decadal birth cohort, and *β*_2_ is a vector of slope parameters for the effect of exposure time, *E*, on fertility for each decadal birth cohort. We control for exposure time (i.e. number of reproductive years) to adjust for individuals who have not completed their reproductive life. We fit this model independently for male and female individuals.

In order to assess how the age at first reproduction of individuals married to cousins and individuals married to non-cousins changed over time (question 5.3), we estimate age at first birth (for individuals born in decades between 1940 and 2000) as a function of decadal birth cohort and marriage type, using a Poisson regression specified by the following set of equations:
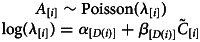

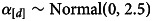

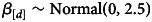
where the response variable *A* is the observed age at first birth of each individual and other terms are as defined above. We fit this model independently for male and female individuals.

### 2.9. Results computation

For each model of interest, we used the rethinking package in R (McElreath, 2020) to compute and analyse the mean and the compatibility intervals (89% PI, percentile interval) of the posterior distribution of each model parameter. We also computed the Type S error, *ps* – the proportion of the posterior distribution on the far side of 0 – which gives the probability that an estimate's sign is reversed (Gelman & Carlin, 2014). We interpret values of *ps* close to 0 (i.e. *ps <* 0.05) as indicative of a reliable non-zero effect, while values closer to 1 indicate a less reliable effect.

## 3. Results

In the following sub-sections, we describe our main results. Table 2 (questions 1–3) and Figure 5 (questions 4 and 5) present a summary of these results. Additional results are provided in the Supplementary Materials, Tables S1–S20.

**Table 2.** Summary of results on frequency of cousin marriage and pool of potential partners. Total sample size of married adults for all these analyses *n* = 1310. The dashed lines separate analyses conducted with different statistical models. The parameters extracted for questions 2.2 and 2.3 are, respectively, the slope (*β*) and intercept (*θ*) coefficients related to the same predictor from the same model. The parameters extracted for question 3.2 pertain to the effects of two predictors in the same model. The estimates for questions 2.2, 2.3, 3.1 and 3.2 are from analyses with standardised predictors

Question	Outcome	Predictor	Mean	Lower PI	Upper PI	*ps*
1.1	Cousin marriage	Birth cohort (*β*)	−0.01	−0.03	0.00	0.091
2.1	Cousin marriage	Number of cousins (*β*)	0.26	0.22	0.30	*<*0.001
2.2	Cousin marriage	Frequency of cousins (*β*)	0.98	0.85	1.12	*<*0.001
2.3	Cousin marriage	Frequency of cousins (*θ*)	0.23	0.21	0.25	*<*0.001
3.1	Frequency of cousins	Birth cohort (*β*)	−0.21	−0.26	−0.17	<0.001
3.2	Cousin marriage	Frequency of cousins (*β*)	1.02	0.89	1.16	*<*0.001
3.2	Cousin marriage	Birth cohort (*β*)	0.16	0.03	0.28	0.027

### 3.1. Frequency of cousin marriage over time

**Figure 1.**
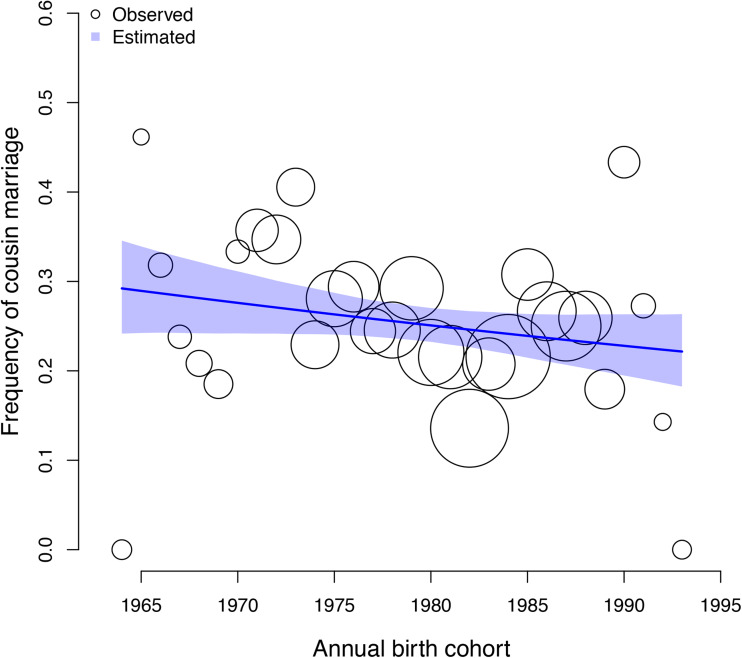
Observed (circles) and predicted (shaded region) frequency of cousin marriage (y axis) for birth-years between 1964 and 1993 (x axis). The shaded region represents the 89% compatibility interval of the predicted distribution. The predicted mean estimate slightly declined from 29% in 1964 to 22% in 1993. The circles represent annual birth-year ‘bins’, within which the empirical frequency of cousin marriage is displayed for illustration purposes; all analyses are based on the individual level data, and the sample size of individuals combined in each bin is indicated by the scale of the circles (ranging from 13 to 88 individuals). The total sample here includes n = 1331 individuals.

The frequency of cousin marriage among Tsimane has slightly decreased over time, from 29% of individuals born in 1964 to 22% of individuals born in 1993, *β*: −0.01 (89% PI −0.03, 0.00; *ps*= 0.091). See Figure 1 for a visualisation of the trend.

### 3.2. Frequency of cousin marriage in relation to the pool of eligible partners

**Figure 2.**
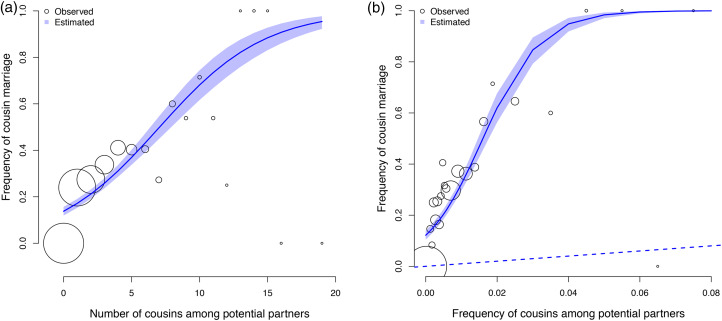
Observed (circles) and predicted (shaded region) frequency of cousin marriage (y axes) as a function of number of cousins available as potential partners (x axis, panel a) and as a function of the frequency of cousins in the pool of potential partners of each individual (x axis, panel b). The shaded regions represent the 89% compatibility intervals of the predicted frequencies. All analyses are based on the individual level data. For illustration, each circle represents a ‘bin’, an x-axis range in which we calculate frequency estimates for illustration purposes. The size of the circles is scaled by sample size (from 2 individuals, for the highest x-axis ranges, to 329 individuals, for the lowest). The dashed line—which has a slope of 1—represents a linear relationship between the frequency of cousin marriage and the frequency of cousins available as partners; it represents the expected level of cousin marriage under random mating.

We find a positive association between the frequency of cousin marriage and the availability of cousins as eligible partners, both in terms of the absolute number of cousins – *β*: 0.26 (89% PI 0.22, 0.30; *ps <* 0.001, Figure 2a) – and the frequency of cousins in the pool of eligible partners of each individual – *β*: 0.98 (89% PI 0.85, 1.12; *ps <* 0.001, Figure 2b). The frequency of cousin marriage among Tsimane is also much higher than the frequency of cousins available at any realised level – e.g. we estimate a 23% chance of cousin marriage (*θ* = 0.23, 89% PI 0.21, 0.25; *ps <* 0.001) even among individuals for whom cousins constitute only 1% of the pool of eligible partners. See Table 2.

### 3.3. Change in the pool of eligible partners over time

**Figure 3.**
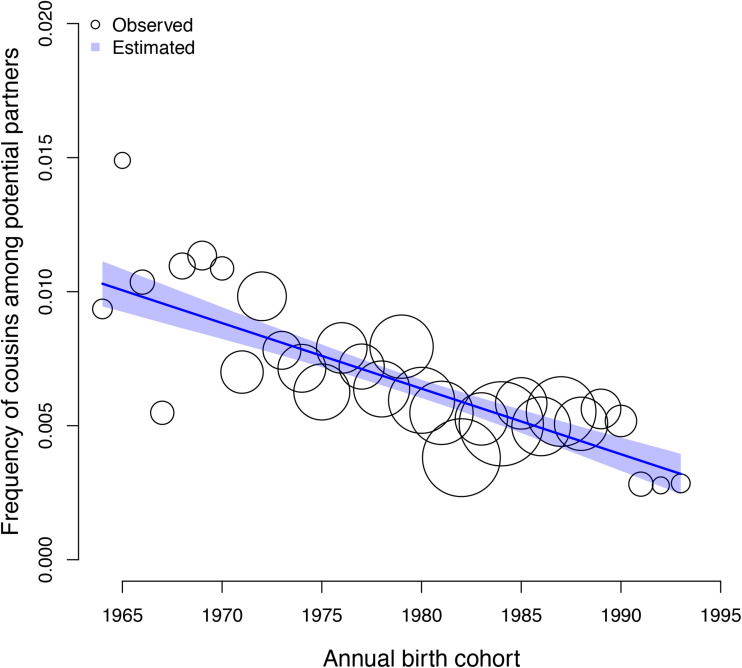
Observed (circles) and predicted (shaded region) frequency of cousins in the pool of potential partners of each individual (y axis) as a function of annual birth cohort (x axis). The shaded region represents the 89% compatibility interval. The predicted mean estimate of frequency of cousins declined from 1% in 1964 to 0.3% in 1993. Each circle represents a ‘bin’, an x-axis averaged frequency estimate for each birth year calculated for illustration purposes. The size of the circles is scaled by sample size (ranging from 13 to 88 individuals).

Individuals born more recently have a higher number of cousins and – to a greater extent – non-cousins available as eligible partners than individuals born in earlier cohorts. However, we find a negative association between the frequency of cousins in the pool of potential partners of a given individual and annual birth cohort, *β*: −0.21 (89% PI −0.26, −0.17; *ps <* 0.001). The expected difference in the frequency of cousins eligible as partners for individuals in the 1964 birth cohort and individuals in the 1993 birth cohort is −0.01 (89% PI −0.01, −0.01; *ps <* 0.001, Figure 3). In the model estimating the association between the frequency of cousin marriage and the frequency of cousins in the pool of potential partners of individuals, controlling for annual birth cohort, we find a positive association between the frequency of cousin marriage and the frequency of cousins as eligible partners, *β*: 1.02 (89% PI 0.89, 1.16; *ps <* 0.001). In this model, the association between the frequency of cousin marriage and annual birth cohort is lower and less reliable, *β*: 0.16 (89% PI 0.03, 0.28; *ps*: 0.027; see Table 2).

### 3.4. Age-specific offspring survival and fertility

**Figure 4.**
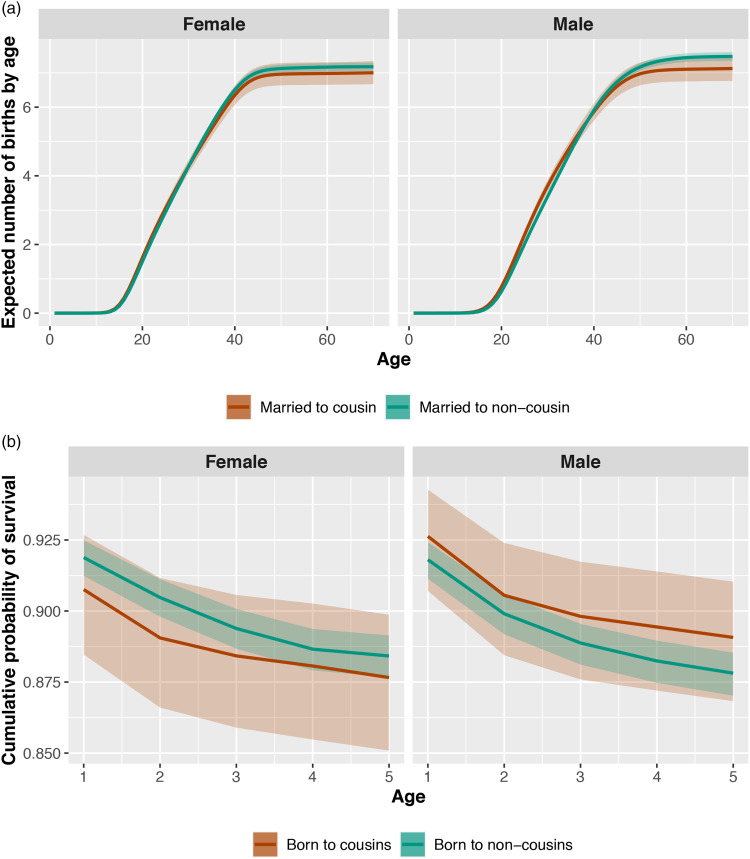
Fertility and offspring survival as a function of cousin marriage. In panel (a) we plot expected number of children (y axis) for each age of life (x axis). In panel (b) we plot the posterior probabilities of cumulative survival (y axis) for each age of life from 0 to 5 (x axis). We plot estimates for female individuals in the left frames and male individuals in the right frames. Estimates are colored by marriage type categories. The bands represent 89% compatibility intervals and reflect the precision of the estimated mean.

Both male and female children born to cousins have similar probability of survival to age 5 compared with children born to non-cousin parents. The difference in survival probability as a function of parental cousin marriage is: for male children, 0.01 (89% PI −0.01, 0.04; *ps* = 0.161); for female children, −0.01 (89% PI −0.03, 0.02; *ps* = 0.328) (Figure 4b). Fertility across the life course is similar for men married to cousins and for men married to non-cousins. The difference in total predicted number of offspring for a man of age 50 is: −0.20 (89% PI −0.54, 0.15; *ps* = 0.178). Fertility is similar for women married to cousins and for women married to non-cousins. The difference in total predicted number of offspring for a woman of age 50 is −0.17 (89% PI −0.50, 0.19; *ps* = 0.224; Figure 4a). Age at first reproduction within cousin marriages is reliably earlier than within non-cousin marriages, both for men – expected difference in estimated years −0.75 (89% PI −1.00, 0.00; *ps <* 0.001) – and for women – expected difference: −0.35 (89% PI −1.00, 0.00; *ps <* 0.001).

### 3.5. Temporal trends in survival and fertility

**Figure 5.**
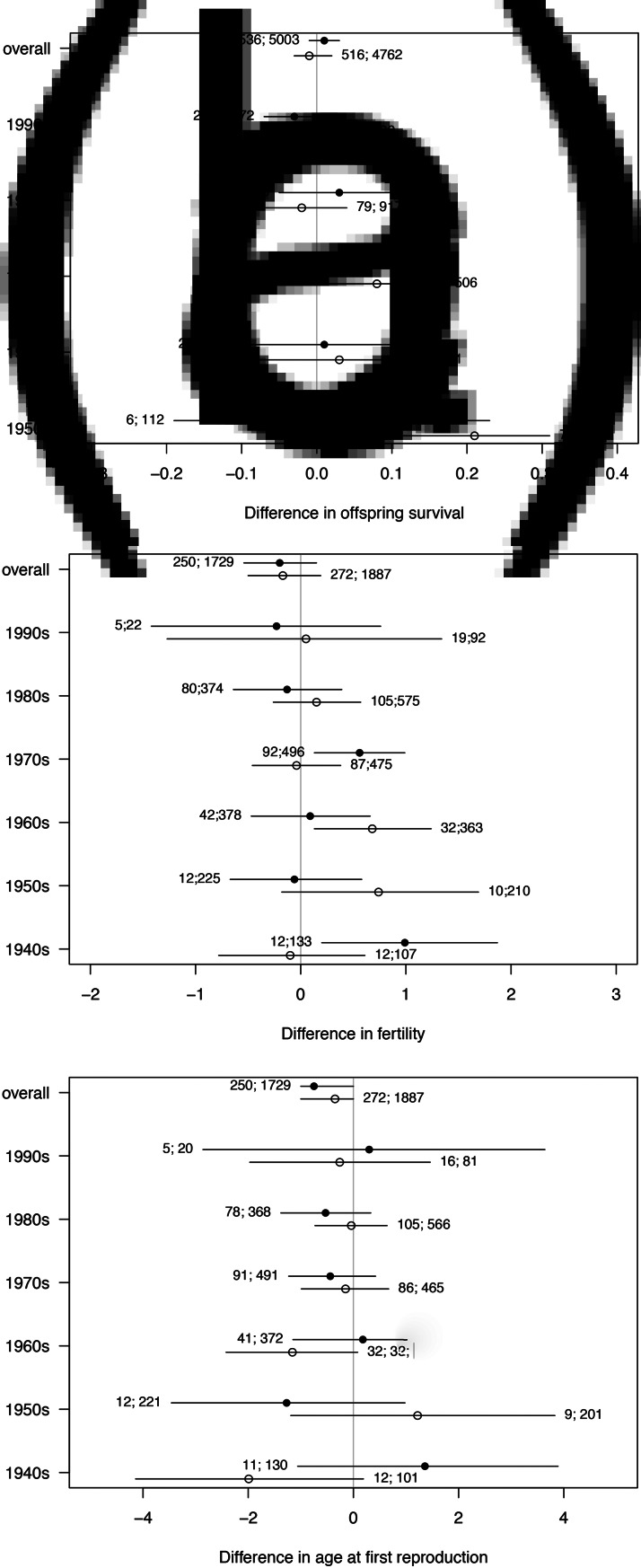
Temporal trends in the association between cousin marriage and fertility/survival. In panel (a), we plot offspring survival outcomes. In panel (b), we plot fertility outcomes. And, in panel (c), we plot age at first reproduction outcomes. Each horizontal bar represents the posterior distribution of the contrasts (i.e., differences) between families practicing cousin marriage and families not practicing it, in each decadal birth cohort (questions 5). Estimates for males are plotted with black circles, estimates for females are plotted with white circles. In the first rows, estimates are averaged across the whole sample (questions 4). The numbers represent the sample sizes of each decadal birth cohort. The first number is the sample size of individuals in families practicing cousin marriage, and the second number is the sample size of individuals in families not practicing cousin marriage.

We find no reliable evidence of differences in survival to age 5 as a function of parental marriage type, for any of the decadal birth cohorts between 1950 and 2000 (see Figure 5a). There are no strong differences in fertility between individuals married to cousins and individuals married to non-cousins in most decades. Only men married to cousins born between 1940 and 1950 and men married to cousins born between 1970 and 1980 have reliably higher fertility than men married to non-cousins born in the same decades. The expected difference in number of children for men born between 1940 and 1950 is 0.99 (89% PI 0.20, 1.87; *ps* = 0.025). The expected difference in number of children for men born between 1970 and 1980 is 0.56 (89% PI 0.13, 0.99; *ps* = 0.018). Also, women married to cousins and born between 1960 and 1970 have higher fertility than women married to non-cousins born in the same decades, with an expected difference in number of children of 0.68 (89% PI 0.13, 1.24; *ps* = 0.024). Slight differences in fertility as a function of cousin marriage apparent in early decades disappear in more recent decades, where we have more observations and thus statistical power (see Figure 5b). We find no strong evidence of temporal trends in age at first reproduction as a function of marriage type for either sex (see Figure 5c).

#### 4. Discussion

### 4.1. Overview

The frequency of cousin marriage has decreased only slightly over time among Tsimane people born between 1964 and 1993 (question 1). The probability of cousin marriage relates to the availability of eligible cousins, both in absolute numbers and relative to non-cousins in the marriage market. Consistent with stated Tsimane cultural preferences, cousin marriage occurs at higher rates than expected by chance (questions 2). While market integration and increasing exposure to Bolivian cultural norms may affect Tsimane marriage practices, we find that changes in the frequency of cousin marriage over time remain tightly connected to the frequency of cousins in the pool of eligible partners (questions 3). Individuals married to cousins exhibit only very slight differences in life histories, with earlier ages of first reproduction, but similar completed fertility and offspring child survival compared with those married to non-cousins (question 4). Analyses on temporal trends in fitness (questions 5) did not reveal clear differences across decadal birth cohorts as a function of marriage type in offspring survival, fertility and age at first reproduction. Minimal differences in fitness visible for individuals born in the early decades tend to disappear for individuals born in more recent decades. Overall, our findings support the prediction that young Tsimane people choose their spouses from a broader pool of potential partners compared to their ancestors. Demographic and socioeconomic shifts – e.g. those that shape kin and non-kin networks, and therefore fitness consequences of different marriage strategies – may lead individuals to use different partner selection criteria. Nevertheless, entrenched cultural traditions may counteract this change and keep the frequency of cousin marriage relatively stable in this population.

### 4.2. Demographic factors shaping the frequency of cousin marriage

The decline in the frequency of cousin marriage – from 29 to 22% – over birth cohorts spanning a period of 30 years has been remarkably modest. This finding recapitulates ethnographic reports describing Tsimane as particularly successful in preserving their cultural identity, for instance by continuing to speak and teach their language, and by maintaining traditional fishing and hunting practices. The slight decline in the frequency of Tsimane cousin marriage, however, accompanies a demographic shift that is reshaping social networks. Individuals born more recently may have more cousins available as potential partners than individuals born earlier, as a result of the increase in fertility among Tsimane people born from the 1930s through the 1970s (see supplement of: Costa et al., 2018). In particular, a drop in mortality rates (Gurven et al., 2007) may have increased the number of cousins in each age class. However, the number of potential partners who are non-cousins increased at a much higher rate in recent years, as a consequence of the population growth rate (Lukas et al., 2005; Gurven et al., 2017). Accordingly, the frequency of cousins in the pool of eligible partners of a given individual has decreased over time (question 3.1). The slight change in the frequency of cousin marriage over time is in fact almost completely associated with the change in the frequency of cousins as eligible partners over time (question 3.2). The remaining effect of birth year, after we account for the changes in frequency of cousins, is negligible. Tsimane people make marriage decisions on the basis of partner availability. The frequency of cousin marriage is constrained by the availability of cousins as eligible partners, However, Tsimane marry cousins at rates higher than what would be expected if individuals chose partners randomly, purely according to the demographic composition of the pool of the eligible partners. The frequency of cousin marriage is in fact higher than what would be predicted from the frequency of marriageable cousins (Hajnal, 1963) (question 2.3). According to our results, when individuals have more than 12 cousins as potential partners, more than 80% of them will marry a cousin. Another potential, although untested, explanation for the slight decrease in the frequency of cousin marriage could be heightened competition among same-sex siblings for marrying a cousin (Chagnon et al., 2017). This competition may arise from the increased number of siblings resulting from population growth. It is important to note, however, that the number of siblings is expected to increase linearly, much like the absolute number of cousins, and therefore any potential effect of this competition should be minimal, because the exponential increase in the number of first cousins is even larger in a growing population.

### 4.3. Fitness consequences of cousin marriage

Overall, the life-history consequences associated with cousin marriage in Tsimane are quite limited. The average relatedness among individuals in the whole population might be relatively high owing to endogamy and because many Tsimane individuals who are not married to a first cousin are nevertheless married to a more distant cousin (Swinford et al., 2023). Socioeconomic factors may complicate the influence of direct biological factors in determining the modest differences between families practising different marriage strategies. This is due to the reported higher prevalence of cousin marriage in more remote areas (unpublished observations reported in: Patel et al., 2007). Fitness differences as a function of marriage type are minimal in the analyses for decadal birth cohorts, and disappear, in particular, for individuals born in more recent decades. The diminishing disparities found in fitness indicators suggest that the socioeconomic conditions of Tsimane people might be changing, possibly becoming more homogeneous across locations and environmental contexts, owing to increasing market integration and acculturation, changes in kinship and social networks, changes in residence and travel patterns, and increasing access to healthcare and contraception. Averaging offspring survival across years, we found no difference between sons and daughters born to cousins and sons and daughters born to non-cousins (question 4.1). In cases when children born to cousins live in remote villages where child mortality rates are higher than in acculturated villages closer to market towns (Gurven et al., 2007), we speculate that potential costs in child mortality from socioeconomic conditions are compensated for by benefits from increased kin support in families practising cousin marriage (Sear & Mace, 2008; Willführ et al., 2018). We were unable to detect stable differences in offspring child survival by decadal birth cohort (question 5.1). This finding is in line with previous research reporting little change in child mortality across all the geographical and social contexts of the Tsimane territory over the second half of the twentieth century (Gurven et al., 2007). The effects of cousin marriage on fertility are very limited (question 4.2). Averaging fertility across the whole sample, we find no differences in fertility as a function of marriage type. As in the survival model, we were unable to detect stable differences in fertility as a function of marriage type by decadal birth cohort (question 5.2). The only differences we detected were higher fertility for cousin-married women born in the 1960s, and higher fertility for cousin-married men born in the 1940s and 1970s. The reproductive advantage found for individuals born in these periods may be explained by improved support from kin in families practising cousin marriage (Hooper et al., 2015; Sear & Coall, 2011) or norms for higher fertility among families practising cousin marriage (Mcallister et al., 2012). However, differences in number of children as a function of marriage type become less marked for the most recent birth cohorts, for whom the estimates are more reliable because of the larger sample size. Kin support may have become less significant in influencing fertility for the younger generations, owing to shifts in kin-based and sharing networks. The fading of differences for individuals born in the decades from 1980 and onward could be also a consequence of a nascent demographic transition – with fertility reduction – across all the areas of the Tsimane territory (Gurven et al., 2017). We observed that both women and men marrying cousins may gain slight fitness benefits in terms of earlier age at first reproduction (question 4.3). This suggests that cousin marriage might ease the problem of finding a mate in this population, especially in the more remote villages where the number of eligible partners can be limited (Cavalli-Sforza et al., 2013). Increased parental control in marriage choices might also be determining the earlier age at first reproduction for individuals in cousin marriage among the Tsimane. In demographic interviews conducted between 2002 and 2005 (M. Gurven, unpublished observations), arranged marriages were four times more likely than non-arranged marriages if the spouses belonged to the cross-lineage. The opportunity to get married earlier can be an incentive to marry a cousin for Tsimane: prestigious Tsimane men marry wives who give birth at earlier ages (Von Rueden et al., 2011). Early age at reproduction is in fact the strongest pathway between fitness and status in Tsimane (Von Rueden et al., 2011). As for the temporal change in trends of age at first reproduction (question 5.3), slight differences found for individuals born in the early decades disappear for individuals born in the most recent decades, probably as a result of the increased availability of non-cousin partners in the more recent period or the reduced risk of adverse outcomes of giving birth at a young age.

### 4.4. Study limitations

The genealogical data used in the current study provide information only on individuals’ birth and death dates and their parents’ identity (see the ‘Methods’ section), because only these data span across several decades. Data do not contain information on individuals’ village of provenance, health or wealth. These details would have been valuable for investigating the impact of socioeconomic factors on the frequency of cousin marriage and its effects on fitness. Another potential data limitation is the absence of information regarding marriages, such as when the couples were married and whether the unions were arranged or not. Our method of constructing family unions is based on shared parentage for a given child (see the ‘Methods’ section). Consequently, we do not include couples who never had children, which could affect fitness estimates if childless couples were more or less likely to be cousins. Moreover, extra-marital offspring may inflate the number of apparent marriages. Because we lack information on the great-grandparents for most couples, we cannot discern patterns related to second cousin marriage, which is also frequent among Tsimane. Nevertheless, the dynamics we identified in first-cousin marriage practice and kinship structure can provide insights into potential dynamics in other types of kin marriages. Our sample is biased towards young individuals (especially in the offspring groups), because we need to know the grandparents of individuals to infer whether they married a cousin or not. For individuals born recently, we do not have data to infer survival and fertility across the lifespan. This limits our ability to use age-structured population projection models to investigate the potential lineage consequences of trade-offs between survival and reproduction (Dalzero et al., 2023). As for the analyses on temporal change in fitness, we could not make strong inferences about individuals born in the decades from 1900 to 1940, because of the low sample size. We used a strict relatedness classification (i.e. including only individuals with eight grandparents known) for the analyses on change in frequency of cousin marriage in relation to the available partners, and a relaxed relatedness classification (i.e. discarding only individuals with eight grandparents unknown) for the analyses on fitness and temporal change in fitness over time. The rationale for this decision was to strike a balance between avoiding the possible confound of classifying people as not having cousins as available partners because their grandparents are unknown, while increasing sample size in the fitness analyses. Some individuals in cousin marriages could be incorrectly classified as in non-cousin marriages under the relaxed condition. Nevertheless, we conducted robustness checks and found that our results qualitatively hold using either approach for the comparisons with a sufficient sample size (code and output files are provided in the repository). The sample might also be biased because genealogical data is likely to mainly include individuals who survived and reproduced, and therefore appear as ancestors. However, this bias applies to both families practising cousin marriage and families not practising it and therefore should not influence our estimation of the differences in survival and fertility.

### 4.5. Conclusion and future directions

Our study of the dynamics of cousin marriage among Tsimane highlights the importance of changes in the composition of the pool of eligible partners. In recent decades, the Tsimane have been choosing their spouses from a broader pool of potential partners. This finding is presumably due to the high population growth rate of Tsimane and the migration of Tsimane into larger and less isolated settlements. However, cousin marriage is practised far more frequently among Tsimane than expected by chance. Cousin marriage among Tsimane is a preferred marriage choice, and this preference, in the examined decades, has been relatively stable. The pace of change may be countered by cultural inertia, as observed in other contexts (e.g. Bashir & Nazir, 2022; Shenk et al., 2016). Cousin marriage entails very modest or negligible fitness consequences in terms of offspring survival and fertility. In contrast, cousin marriage seems to have given Tsimane people some advantages on the marriage market, easing the marriage process in this population, as suggested by the earlier age at first reproduction of cousin-married individuals. However, any detectable differences in the examined traits between individuals practising cousin marriage and not practising it have disappeared closer to the present. This reduction in fitness differences by marriage type suggests a homogenization owing to increasing acculturation, healthcare access and the use of contraception, and social connectedness across the Tsimane territory. Accordingly, the factors that have guided marriage choice and led to a strong preference for cousins as spouses (e.g. increased marriage opportunities and kin-based support for those choosing cousins) might also be changing. This change might contribute to a sharper reduction in the frequency of cousin marriages in the future, as has been observed in other societies undergoing market integration (Bittles & Black, 2010).

In order to better understand dynamics in the frequency of cousin marriage among Tsimane, future research could explore the impact of cousin marriage on social networks, economic production and health. One interesting angle could be to investigate how cousin marriage can persist at rates higher than what we could expect considering the demographic and socioeconomic changes. This persistence is also observable in other cultures, including immigrants adapting to entirely new socioeconomic conditions (Reniers, 2001; Shaw, 2001). An analysis of the regional variation in cousin marriage across the Tsimane territory, which quantifies its frequency both near market towns and in the most remote areas, would help to investigate the socioeconomic correlates of this practice. Further research could also explore how cultural traits such as religious beliefs determine marital choices. Our analyses reveal the complexity of the potential connections between cultural changes in marriage practices and the underlying societal changes that shape partner availability and fitness returns to different marriage choices. More broadly, our approach showcases a path to use longitudinal data to gain insights into how sociodemographic changes can lead to behavioural changes in human societies.

## Supporting information

Dalzero et al. supplementary material Dalzero et al. supplementary material

## Data Availability

Code for reproducing our methods and results can be accessed on the Open Science Framework: https://doi.org/10.17605/OSF.IO/PK8F7. Individual-level data are stored in the Tsimane Health and Life History Project (THLHP) Data Repository, and are available through controlled access protocols (for ethical reasons related to data sovereignty). The THLHP's highest priority is the safeguarding of human subjects and minimisation of risk to study participants. The THLHP adheres to the CARE Principles for Indigenous Data Governance, which assure that the Tsimane: (1) have sovereignty over how data are shared; (2) are the primary gatekeepers determining ethical use; (3) are actively engaged in the data generation; and (4) derive benefit from data generated and shared whenever possible. The THLHP is also committed to the FAIR Principles to facilitate data use. Requests for individual-level data should take the form of an application that minimally details the exact uses of the data and the research questions to be addressed, procedures that will be employed for data security and individual privacy, potential benefits to the study communities, and procedures for assessing and minimising stigmatising interpretations of the research results (see the following webpage for links to the data sharing policy and data request forms: https://tsimane.anth.ucsb.edu/data.html). Requests for individual-level data will require institutional approval and will be reviewed by an Advisory Council composed of tribal leaders, tribal community members, Bolivian scientists and the THLHP leadership. The study authors and the THLHP leadership are committed to open science and are available to assist interested investigators in preparing data access requests.

## Supplementary material

To view supplementary material for this article, please visit https://doi.org/10.1017/ehs.2024.11
