# Supplementary material for: Cross-cousin marriage among Tsimane forager–horticulturalists during demographic transition and market integration
Source: Evol Hum Sci. 2024 Mar 26;6:e18. doi: 10.1017/ehs.2024.11 (PMC10988167; doi:10.1017/ehs.2024.11)
Supplement: Dalzero et al. supplementary material [file S2513843X24000112sup001.pdf]

Supplementary Materials for: "Cross-cousin marriage among Tsimane forager-horticulturalists during demographic transition and market integration".

Arianna Dalzero, Bret A. Beheim, Hillard Kaplan, Jonathan Stieglitz, Paul L. Hooper, Cody Ross, Michael Gurven, Dieter Lukas

December 2023

1 Tables S1-S20 showing coefficient results and sample sizes of the analyses.

Table S1: Frequency of cousin marriage over time.

parameter	mean	low PI	up PI	ps
Beta	-0.01	-0.03	0.00	0.091
Odds ratio	0.99	0.97	1.00	0.091

Table 1: Mean (second column), 5% lower (third column) and 94% upper boundary (fourth column) of the 89% percentile interval of the posterior probability distribution, and Type S error (fifth column) of the β coefficient (first row), and odds ratio (second row) for the frequency of cousin marriage as a function of the individuals' annual birth cohort.

Table S2: Frequency of cousin marriage as a function of number of cousins eligible partners.

parameter	mean	low PI	up PI	ps
Beta	0.26	0.22	0.30	<0.001
Odds ratio	1.30	1.25	1.35	<0.001

Table 2: Mean (second column), 5% lower (third column) and 94% upper boundary (fourth column) of the 89% percentile interval of the posterior probability distributions, and Type S error (fifth column), of the β coefficient (first row), and odds ratio (second row) for the frequency of cousin marriage as a function of the individuals' number of cousins as eligible partners.

Table S3: Frequency of cousin marriage as a function of frequency of cousins eligible partners.

parameter	mean	low PI	up PI	ps
Beta	1.24	1.07	1.41	<0.001
Odds ratio	1.00	1.00	1.00	<0.001
Intercept	0.50	0.50	0.50	<0.001

Table 3: Mean (second column), 5% lower (third column) and 94% upper boundary (fourth column) of the 89% percentile interval of the posterior probability distributions, and Type S error (fifth column), of the β coefficient (first row), odds ratio (second row), and θ coefficient (i.e. intercept, third row), for the frequency of cousin marriage as a function of the individuals' frequency of cousins among the eligible partners.

Table S4: Frequency of cousin marriage as a function of (standardized) frequency of cousins eligible partners (same model as above with standardized predictor for easier interpretation of results).

parameter	mean	low PI	up PI	ps
Beta	0.98	0.85	1.12	<0.001
Odds ratio	2.68	2.33	3.07	<0.001
Intercept	0.23	0.21	0.25	<0.001

Table 4: Mean (second column), 5% lower (third column) and 94% upper boundary (fourth column) of the 89% percentile interval of the posterior probability distributions, and Type S error (fifth column), of the β coefficient (first row), odds ratio (second row), and θ coefficient (i.e. intercept, third row), for the frequency of cousin marriage as a function of the individuals' (standardized) frequency of cousins among the eligible partners. These results are reported in the manuscript.

Table S5: Frequency of cousins as eligible partners as a function of annual birth cohort.

parameter	mean	low PI	up PI	ps
Beta	-0.0002	-0.0003	-0.0002	<0.001
Contrast mu	-0.01	-0.01	-0.01	<0.001

Table 5: Mean (second column), 5% lower (third column) and 94% upper boundary (fourth column) of the 89% percentile interval of the posterior distributions, and Type S error (fifth column) of the β coefficient (first row), and probability difference (contrast) between the earliest and last recorded individuals' birth cohort (second row), for frequency of cousin as eligible partners as a function of individuals' annual birth cohort.

Table S6: Standardized frequency of cousins as eligible partners as a function of (standardized) annual birth cohort (same model as above with standardized predictors for easier interpretation of results).

parameter	mean	low PI	up PI	ps
Beta	-0.21	-0.26	-0.17	<0.001
Contrast mu	-0.01	-0.01	-0.01	<0.001

Table 6: Mean (second column), 5% lower (third column) and 94% upper boundary (fourth column) of the 89% percentile interval of the posterior distributions, and Type S error (fifth column) of the β coefficient (first row), and probability difference in μ (contrast) between the earliest and last recorded individuals' birth cohort (second row), for (standardized) frequency of cousin as eligible partners as a function of (standardized) individuals' annual birth cohort. These results are reported in the manuscript.

Table S7: Frequency of cousin marriage as a function of the frequency of cousins, stratified by annual birth cohort.

parameter	mean	low PI	up PI	ps
Beta frequency	1.29	1.11	1.47	<0.001
Odds ratio frequency	1.00	1.00	1.00	<0.001
Beta birth year	0.02	0.01	0.04	0.014
Odds ratio birth year	1.02	1.01	1.04	0.014

Table 7: Mean (second column), 5% lower (third column) and 94% upper boundary (fourth column) of the 89% percentile interval of the posterior distributions, and Type S error (fifth column), of the β coefficient and odds ratio, for chance of cousin marriage as a function of the proportion of cousins, stratified by the individuals' annual birth cohort. The beta coefficient and odds ratio estimate the effect of the frequency of cousins (row 1-2) and annual birth cohort (row 3-4).

Table S8: Frequency of cousin marriage as a function of the (standardized) frequency of cousins, stratified by (standardized) annual birth cohort (same model as above with standardized predictors for easier interpretation of results).

parameter	mean	low PI	up PI	ps
Beta frequency	1.02	0.89	1.16	<0.001
Odds ratio frequency	2.79	2.43	3.20	<0.001
Beta birth year	0.16	0.03	0.28	0.027
Odds ratio birth year	1.17	1.03	1.32	<0.001

Table 8: Mean (second column), 5% lower (third column) and 94% upper boundary (fourth column) of the 89% percentile interval of the posterior distributions, and Type S error (fifth column), of the β coefficient and odds ratio, for chance of cousin marriage as a function of the (standardized) proportion of cousins, stratified by the (standardized) individuals' annual birth cohort. The β coefficient and odds ratio estimate the effect of frequency of cousins (row 1-2) and annual birth cohort (row 3-4). These results are reported in the manuscript.

Table S9: Sample size by annual birth cohort for the analyses on frequency of cousin marriage.

birth year	sample size
1964	17
1965	13
1966	22
1967	21
1968	24
1969	27
1970	21
1971	42
1972	49
1973	37
1974	48
1975	57
1976	51
1977	45
1978	57
1979	65
1980	68
1981	65
1982	81
1983	53
1984	88
1985	52
1986	60
1987	72
1988	54
1989	39
1990	30
1991	22
1992	14
1993	16

Table 9: Sample size of married adults (second column) for each birth year (first column), for all the analyses on frequency of cousin marriage and eligible partners over time (tables above). Total sample size n = 1331.

24

Table S10: Offspring children age-specific survival up to age 5.

marriage	sex	state	mean	low PI	up PI
Cousin	Male	Born	0.89	0.87	0.91
Cousin	Female	Born	0.88	0.85	0.90
Non-cousin	Male	Born	0.88	0.87	0.89
Non-cousin	Female	Born	0.88	0.87	0.89

Table 10: Mean (fourth column), 5% lower (fifth column) and 94% upper boundary (sixth column) of the 89% percentile interval of the posterior distributions of the sub-populations, male and female (second column), born (third column) in families practicing cousin marriage or not (first column), for cumulative probability of survival up to age 5.

Table S11: Offspring children age-specific survival up to age 5 contrasts

sex	state	mean	low PI	up PI	ps
Male	Born	0.01	-0.01	0.03	0.186
Female	Born	-0.01	-0.03	0.02	0.312

Table 11: Mean (third column), 5% lower (fourth column) and 94% upper boundary (fifth column) of the 89% percentile interval and Type S error (sixth column) of the posterior distribution for the contrasts (difference) between the posterior probability distributions of sub-populations, male and female (first column), offspring (second column) of families practicing cousin marriage versus not practicing it (e.g., male individuals born to cousins versus male individuals born to unrelated parents), for cumulative probability of survival up to age 5.

Table S12: Offspring children age-specific survival up to age 5 sample sizes for each age.

	cousin male	non-cousin male	cousin female	non-cousin female
1	536	5003	516	4762
2	478	4510	453	4308
3	458	4287	424	4126
4	438	4084	404	3946
5	421	3905	380	3775

Table 12: Total sample size (number of individuals 'at risk') in the ages of life from 1 to 5, for offspring of cousin parents and offspring of non-cousin parents.

Table S13: Age-specific fertility.

marriage	sex	state	mean	low PI	up PI
Cousin	Male	Married	6.97	6.64	7.30
Cousin	Female	Married	6.96	6.64	7.29
Non-cousin	Male	Married	7.17	7.06	7.29
Non-cousin	Female	Married	7.13	7.01	7.24

Table 13: Mean (fourth column), 5% lower (fifth column) and 94% upper boundary (sixth column) of the 89% percentile interval of the posterior probability distributions of the sub-populations, male and female (second column), married (third column) to cousins or not (first column), for cumulative fertility in reproductive ages (12-50).

Table S14: Age-specific fertility contrasts.

sex	state	mean	low PI	up PI	ps
Male	Married	-0.20	-0.54	0.15	0.178
Female	Married	-0.17	-0.50	0.19	0.224

Table 14: Mean (third column), 5% lower (fourth column) and 94% upper boundary (fifth column) of the 89% percentile interval and Type S error (sixth column) of the posterior probability distribution for the contrasts (difference) between the posterior probability distributions of sub-populations male and female (first column), married (second column) to cousins and not (e.g., male individuals married to cousins versus male individuals married to non-cousin partners) for cumulative fertility in the reproductive ages (12-50).

Table S15: Age at first reproduction (from age-specific fertility).

marriage	sex	state	mean	low PI	up PI
Cousin	Male	Married	21.25	21.00	22.00
Cousin	Female	Married	18.65	18.00	19.00
Non-cousin	Male	Married	22.00	22.00	22.00
Non-cousin	Female	Married	19.00	19.00	19.00

Table 15: Mean (fourth column), 5% lower (fifth column) and 94% upper boundary (sixth column) of the 89% percentile interval of the posterior probability distributions of the sub-populations, male and female (second column), married (third column) to cousins or not (first column), for age at first reproduction. Age at first reproduction is defined here as the first age when the cumulative probability of fertility exceeds 1.

Table S16: Age at first reproduction (from age-specific fertility) contrasts.

sex	state	mean	low PI	up PI	ps
Male	Married	-0.75	-1.00	0.00	<0.001
Female	Married	-0.35	-1.00	0.00	<0.001

Table 16: Mean (third column), 5% lower (fourth column) and 94% upper boundary (fifth column) of the 89% percentile interval and fraction above 0 (sixth column) of the posterior probability distribution for the contrasts (subtraction difference) between the posterior probability distributions of sub-populations male and female (first column), married (second column) to cousins and not (e.g., male individuals married to cousins versus male individuals married to non-cousin partners) for age at first reproduction. Age at first reproduction is defined here as the first age when the cumulative probability of fertility exceeds 1.

Table S17: Sample size for each reproductive age 12-50 (for age specific fertility and age at first reproduction).

	cousin male	non-cousin male	cousin female	non-cousin female
12	250	1729	272	1887
13	250	1728	272	1887
14	250	1728	272	1887
15	250	1727	272	1883
16	250	1724	268	1869
17	249	1720	268	1855
18	249	1716	265	1830
19	246	1704	262	1796
20	240	1690	255	1754
21	229	1670	251	1702
22	227	1645	246	1655
23	219	1618	233	1610
24	213	1573	218	1568
25	206	1534	203	1507
26	201	1493	191	1449
27	192	1452	182	1376
28	186	1402	167	1314
29	176	1347	158	1244
30	170	1298	149	1177
31	165	1242	138	1106
32	155	1187	130	1061
33	143	1140	122	1007
34	129	1095	113	963
35	125	1040	103	919
36	117	984	94	877
37	110	945	88	837
38	100	889	84	793
39	91	853	77	757
40	84	803	73	702
41	76	762	67	678
42	70	733	60	636
43	63	702	54	591
44	59	669	49	559
45	54	639	43	529
46	51	609	39	493
47	45	566	37	459
48	41	531	33	431
49	35	496	33	402
50	31	473	30	366

Table 17: Total sample size (number of individuals 'at risk') in the reproductive ages of life from 12 to 50, for male and female individuals in cousin marriage and in a marriage with non-cousins.

Table S18: Contrasts of offspring survival by decadal birth cohort.

sex	mean	low PI	up PI	ps	sample size	decade
Male	-0.03	-0.32	0.13	0.589	cousin born: 1; not cousin born: 8	1
Male	0.05	-0.44	0.32	0.308	cousin born: 1; not cousin born: 17	2
Male	-0.06	-0.40	0.17	0.426	cousin born: 4; not cousin born: 40	3
Male	0.06	-0.19	0.23	0.294	cousin born: 6; not cousin born: 112	4
Male	0.01	-0.11	0.10	0.393	cousin born: 23; not cousin born: 260	5
Male	0.06	-0.02	0.13	0.112	cousin born: 42; not cousin born: 530	6
Male	0.03	-0.05	0.10	0.270	cousin born: 59; not cousin born: 943	7
Male	-0.03	-0.07	0.01	0.138	cousin born: 212; not cousin born: 1672	8
Female	-0.09	-0.66	0.31	0.500	cousin born: 0; not cousin born: 2	1
Female	-0.12	-0.70	0.22	0.498	cousin born: 0; not cousin born: 14	2
Female	-0.12	-0.74	0.10	0.502	cousin born: 0; not cousin born: 43	3
Female	0.21	0.05	0.31	0.028	cousin born: 7; not cousin born: 97	4
Female	0.03	-0.09	0.12	0.310	cousin born: 25; not cousin born: 241	5
Female	0.08	-0.02	0.14	0.078	cousin born: 28; not cousin born: 506	6
Female	-0.02	-0.09	0.04	0.343	cousin born: 79; not cousin born: 917	7
Female	-0.03	-0.07	0.01	0.124	cousin born: 167; not cousin born: 1600	8

Table 18: Mean (second column), 5% lower (third column) and 94% upper boundary (fourth column) of the 89% percentile interval of the posterior probability distribution, and Type S error (fifth column), for the contrasts (subtraction difference) between the posterior probability distributions of sub-populations male and female (first column) born in eight decades from 1920 to 2000 (seventh column), in families practicing cousin marriage or not (e.g., male individuals born to cousins versus male individuals born to non-cousin partners), for child survival up to age 5 as a function of individuals' decades of birth. In the sixth column, sample sizes for each sub-population in each decadal birth cohort. The estimates are related to the priors for birth decades where sample size is 0 and we do not consider these decades in the manuscript. In the manuscript, we consider decadal birth cohorts from 1950 to 2000 (decades 4-8).

34

Table S19: Contrasts of fertility by decadal birth cohort.

sex	mean	low PI	up PI	ps	sample size	decade
Male	-1.05	-3.29	0.03	0.060	cousin married: 1; not cousin married: 8	1
Male	327.87	-14.56	312.57	0.493	cousin married: 0; not cousin married: 8	2
Male	2.13	0.55	4.14	0.007	cousin married: 2; not cousin married: 28	3
Male	0.23	-1.18	1.74	0.425	cousin married: 4; not cousin married: 63	4
Male	0.99	0.20	1.87	0.025	cousin married: 12; not cousin married: 133	5
Male	-0.06	-0.67	0.58	0.430	cousin married: 12; not cousin married: 225	6
Male	0.09	-0.47	0.66	0.390	cousin married: 42; not cousin married: 378	7
Male	0.56	0.13	0.99	0.018	cousin married: 92; not cousin married: 496	8
Male	-0.13	-0.64	0.39	0.334	cousin married: 80; not cousin married: 374	9
Male	-0.23	-1.42	0.76	0.377	cousin married: 5; not cousin married: 22	10
Female	120.45	-9.15	182.42	0.496	cousin married: 0; not cousin married: 1	1
Female	-6.18	-15.88	-0.49	0.040	cousin married: 1; not cousin married: 7	2
Female	45.47	-3.51	160.24	0.488	cousin married: 0; not cousin married: 22	3
Female	-0.25	-1.18	0.81	0.326	cousin married: 7; not cousin married: 46	4
Female	-0.10	-0.78	0.61	0.394	cousin married: 12; not cousin married: 107	5
Female	0.74	-0.18	1.69	0.096	cousin married: 10; not cousin married: 210	6
Female	0.68	0.13	1.24	0.024	cousin married: 32; not cousin married: 363	7
Female	-0.04	-0.46	0.38	0.431	cousin married: 87; not cousin married: 475	8
Female	0.15	-0.26	0.57	0.283	cousin married: 105; not cousin married: 575	9
Female	0.05	-1.27	1.34	0.456	cousin married: 19; not cousin married: 92	10

Table 19: Mean (second column), 5% lower (third column) and 94% upper boundary (fourth column) of the 89% percentile interval of the posterior probability distribution, and Type S error (fifth column), for the contrasts (difference) between the posterior probability distributions of sub-populations male and female (first column) born in ten decades from 1900 to 2000 (seventh column), married to cousins or not (e.g., male individuals married to cousins versus male individuals married to non-cousin partners), for total number of children as a function of individuals' decades of birth. In the sixth column, sample size for each sub-population in each decadal birth cohort. The estimates are related to the priors for birth decades where sample size is 0 and we do not consider these decades in the manuscript. In the manuscript, we consider decadal birth cohorts from 1940 to 2000 (decades 5-10).

Table S20: Contrasts in age at first reproduction by decadal birth cohort.

sex	mean	low PI	up PI	ps	sample size	decade
Male	0.63	-7.92	10.05	0.472	cousin married: 1; not cousin married: 8	1
Male	432.79	-26.84	1409.72	0.503	cousin married: 0; not cousin married: 8	2
Male	-1.92	-7.19	3.84	0.268	cousin married: 2; not cousin married: 26	3
Male	-2.10	-5.85	2.02	0.194	cousin married: 4; not cousin married: 62	4
Male	1.36	-1.06	3.89	0.184	cousin married: 11; not cousin married: 130	5
Male	-1.27	-3.46	0.98	0.174	cousin married: 12; not cousin married: 221	6
Male	0.18	-1.15	1.43	0.412	cousin married: 41; not cousin married: 372	7
Male	-0.44	-1.23	0.42	0.194	cousin married: 91; not cousin married: 491	8
Male	-0.53	-1.38	0.33	0.157	cousin married: 78; not cousin married: 368	9
Male	0.30	-2.86	3.64	0.451	cousin married: 5; not cousin married: 20	10
Female	501.37	-21.97	1135.55	0.505	cousin married: 0; not cousin married: 1	1
Female	0.89	-5.71	8.72	0.454	cousin married: 1; not cousin married: 6	2
Female	559.49	-18.42	1572.04	0.500	cousin married: 0; not cousin married: 19	3
Female	-0.64	-3.36	2.18	0.346	cousin married: 7; not cousin married: 41	4
Female	-1.99	-4.14	0.19	0.078	cousin married: 12; not cousin married: 101	5
Female	1.22	-1.19	3.83	0.214	cousin married: 9; not cousin married: 201	6
Female	-1.16	-2.42	0.08	0.070	cousin married: 32; not cousin married: 357	7
Female	-0.15	-0.99	0.67	0.378	cousin married: 86; not cousin married: 465	8
Female	-0.04	-0.73	0.64	0.470	cousin married: 105; not cousin married: 566	9
Female	-0.26	-1.97	1.46	0.399	cousin married: 16; not cousin married: 81	10

Table 20: Mean (second column), 5% lower (third column) and 94% upper boundary (fourth column) of the 89% percentile interval of the posterior probability distribution, and Type S error (fifth column) for the contrasts (difference) between the posterior probability distributions of sub-populations male and female (first column) born in ten decades from 1900 to 2000 (seventh column), married to cousins or not (e.g., male individuals married to cousins versus male individuals married to non-cousin partners), for age at first child as a function of individuals' decades of birth. In the sixth column, sample size for each sub-population in each decadal birth cohort. The estimates are related to the priors for birth decades where sample size is 0 and we do not consider these decades in the manuscript. In the manuscript, we consider decadal birth cohorts from 1940 to 2000 (decades 5-10).
